# Principles of Surgical Treatment of Soft Tissue Sarcomas

**DOI:** 10.3390/cancers17030401

**Published:** 2025-01-25

**Authors:** Marcos R. Gonzalez, Carolina Mendez-Guerra, Megan H. Goh, Juan Pretell-Mazzini

**Affiliations:** 1 Division of Orthopaedic Oncology, Department of Orthopaedic Surgery, Massachusetts General Hospital, Harvard Medical School, Boston, MA 02115, USA; mgonzalez52@mgh.harvard.edu (M.R.G.); mgoh3@mgh.harvard.edu (M.H.G.); 2 Facultad de Ciencias de la Salud, Universidad Peruana de Ciencias Aplicadas, Lima 15067, Peru; mendezguerra.ci@gmail.com; 3 Division of Orthopedic Oncology, Miami Cancer Institute, Baptist Health System South Florida, Plantation, FL 33324, USA; 4 Department of Orthopaedic Surgery, Herbert Wertheim College of Medicine, Florida International University, Miami, FL 33199, USA

**Keywords:** soft tissue sarcoma, surgical management, oncology

## Abstract

Soft tissue sarcoma (STS) is a group of highly heterogeneous tumors of mesenchymal origin that have variable primary site locations and clinical behavior. Despite the broad diversity of STS, the standard of care involves surgical resection with or without radiation therapy (RT) to control local recurrence and systemic treatment in select cases. The complexities of STS require a critical understanding of the preoperative work-up process, surgical treatment, and postoperative management. Advanced imaging plays a vital role in the characterization of the soft tissue mass, preoperative biopsy planning, and disease staging. Surgical treatment prioritizes wide resection with negative margins, supported by newer margin classification systems for better prognosis. Further, advancements in surgical technique have enabled limb-salvage surgery to largely replace amputation in the management of these tumors. Additional surgical considerations, such as nerve preservation, vascular reconstruction, and complex tissue closure, further highlight the complexity of STS management. Lastly, postoperative follow-up is critical for the early detection of local or distant recurrences. For complex cases, such as unplanned excisions or invasive tumors, strategies like re-resection may be beneficial. Ongoing research into imaging, chemotherapy, and targeted therapies will further refine management strategies, especially in complex and recurrent cases. This review highlights the essential aspects of STS surgical management and underscores the need for coordinated, multidisciplinary care to enhance both survival and quality of life for affected patients.

## Simple Summary

Soft tissue sarcoma (STS) is a broad group of rare tumors that occur in the soft tissues. It is challenging to treat STS because they are a diverse cohort of tumors that can occur anywhere in the body, and each can respond differently to treatment. The primary treatment for STS is surgical resection, augmented with other therapies such as radiation therapy to reduce the chances of tumor recurrence, and chemotherapy for high-risk patients. Further, improvements in surgical technique have enabled limb-salvage surgery to replace amputation as the standard of care. Orthopaedic oncologic surgeons serve an important role in the multidisciplinary care of a complex disease like STS. Thus, it is critical to understand each phase of the treatment process. This article provides an in-depth overview of the preoperative workup and considerations, surgical treatment, and postoperative management of patients with STS.

## 1. Introduction

Soft tissue sarcoma (STS) represents a diverse category of rare, mesenchymal-origin tumors, comprising over 50 distinct histologic subtypes that vary in primary site location and clinical behavior [1,2,3]. Globally, STS incidence is estimated to range between 1.8 to 5 cases per 100,000 individuals per year [4]. According to the National Cancer Institute, there are approximately 13,590 new cases annually in the United States alone, leading to an estimated 5200 deaths each year [5]. While most STS cases are diagnosed at a localized, non-metastatic stage, 40–50% of patients will ultimately progress to metastatic disease, which is associated with a notably poor prognosis [6,7,8].

Despite the considerable histologic, anatomic, and clinical variability in STS, treatment protocols have remained largely unchanged over the past 30 years [1], with surgical resection as the gold standard, augmented with radiation therapy (RT) as needed for local tumor control [9]. Achieving negative margins is crucial, as the most reliable predictor of local recurrence is the presence of positive or uncertain surgical margins [10,11]. In fact, insufficient or positive margins are associated with an 80–90% local recurrence rate [12]. Although chemotherapy is well-established in the treatment of advanced STS [13,14], its role in localized STS remains controversial with studies showing mixed results regarding its benefits [15,16,17].

Given the complexity of STS management, a multidisciplinary approach is increasingly critical with orthopaedic oncology surgeons playing a pivotal role. This literature review aims to provide a comprehensive overview of the surgical treatment for the broad, heterogenous disease of STS (except pediatric rhabdomyosarcomas), covering key considerations in preoperative management, surgical intervention, and the postoperative period, as well as addressing challenging situations in STS care.

## 2. Preoperative Workup and Treatment

### 2.1. Importance of Preoperative Evaluation in STS

With the advent of new surgical procedures over the last decades, limb-salvage surgery (LSS) has largely replaced amputation as the standard of care in the management of STS [11]. These advances have prompted the establishment of a comprehensive preoperative evaluation, tailored to each patient’s individual characteristics [18]. In this regard, differences have been found in the rates of preoperative primary site imaging and biopsy between patients with wide excision and those with incomplete excision [19]. Furthermore, only a minority of patients referred for definite re-excision following incomplete tumor resection undergo appropriate preoperative assessments, despite the negative impact of this practice on oncological outcomes [19]. These findings further support the importance of an adequate preoperative assessment in the management of patients with STS, as it greatly influences patient morbidity and mortality. Moreover, several prognostic factors for metastasis and survival should be assessed during the preoperative evaluation of a patient with suspected or confirmed STS. These key prognostic markers include a progressive increase in tumor size, which directly impacts mortality, sarcoma subtype, and histologic grade, with the latter being the most important factor for predicting metastasis [20]. A comprehensive preoperative evaluation requires a multidisciplinary approach, as the input of several specialists is essential for effective decision-making and an adequate treatment strategy [21].

### 2.2. Imaging Modalities for Primary Tumor Assessment

Conventional radiography is recommended for the initial assessment of soft tissue masses in accordance with the appropriateness criteria outlined by the American College of Radiology; alternatively, if the soft-tissue mass is deemed to be clinically superficial, ultrasonography can be utilized [22]. Further investigation using magnetic resonance imaging (MRI) should be conducted in cases of suspected malignancy or cases of inconclusive ultrasound findings [23]. MRI is the imaging of choice for characterizing STS, due to its superior resolution and multiplanar imaging capability in assessing soft-tissue masses [24]. Moreover, this imaging modality is of paramount importance for surgical planning as it provides accurate location, architecture, and vascularization of the primary tumor, as well as characterization of the anatomical structures surrounding the lesion [25]. This imaging technique also plays a critical role in planning the preoperative biopsy, anticipating the histologic grade of the tumor, and deciding the most suitable treatment strategy for each patient [26]. Major pre-operative MRI findings such as peritumoral enhancement [27,28] and the “tail sign” [27,29] in specific STS such as myxofibrosarcoma have important prognostic value, as they indicate macroscopic infiltration and the potential need for more extensive resection to achieve negative surgical margins [30]. Critically, preoperative MRI can be utilized to establish baseline dimensions of the soft tissue mass. The most frequently used guideline of single dimensional assessment of tumors was established by the Response Evaluation Criteria in Solid Tumor (RECIST) working group [31], with the longest diameter of the tumor serving as a strong prognostic indicator in STS [32]. The sum of diameters, product of diameters, and volume are other dimensional tumor assessments that can be done, but their association with prognostication has not been as well-established [32]. Anticipation of the histologic grade of the tumor using a diagnostic MRI score has been recently proposed in the literature [33]. This scoring system is based on morphological MRI features including tumor heterogeneity, and intratumoral and peritumoral enhancement [27]. Furthermore, the use of multiparametric MRI (mpMRI) in assessing early response following neoadjuvant radiotherapy (RT) has also been recently studied, as early response to therapy has been predictive of survival, local recurrence, and distant metastasis [34,35]. Despite the need for further investigation, preliminary results show that the use of mpMRI is feasible in patients with STS receiving neoadjuvant RT [34]. Conversely, the use of contrast-enhanced computed tomography (CT) is the preferred imaging modality in cases of suspected retroperitoneal sarcoma [24]. Likewise, it is also appropriate when MRI is not available or contraindicated [18].

### 2.3. Disease Staging

Disease staging in STS is assessed using both the American Joint Committee on Cancer (AJCC) classification and the Fédération Nationale des Centres de Lutte Contre Le Cancer (FNCLCC) criteria. The AJCC classification provides a tumor, node, and metastasis (TNM) classification [36], whereas the FNCLCC criteria use histological features, such as tumor differentiation, mitotic activity, and extent of necrosis, for tumor grading [37,38]. The lungs are the primary site of metastasis in STS [39]. Therefore, all patients with confirmed disease must be evaluated for pulmonary metastasis prior to definite treatment. The exclusion of secondary lesions in the lungs is typically performed by using a chest CT [40]. In the upcoming lines, current recommendations regarding imaging sites and modalities according to the sarcoma subtype will be outlined. Due to the higher risk of soft-tissue and visceral metastases in patients diagnosed with myxoid liposarcoma, current guidelines recommend the routine incorporation of abdominal and pelvic CT scans in the initial staging of these patients [40]. Moreover, a whole-body MRI may also be considered in selected patients [41]. In cases of clear cell sarcoma, angiosarcoma, or epithelioid sarcoma, regional lymph node assessment is recommended due to the increased risk of nodal invasion [40]. Additionally, due to the increased risk of brain metastasis in patients with alveolar soft part sarcomas [42,43] or clear cell sarcomas, contrast-enhanced CT or MRI of the brain is recommended [40]. Positron emission tomography (PET)-CT has emerged as a complementary diagnostic tool in the initial staging of soft tissue masses, as it has demonstrated effective detection of lymph node metastasis with high sensitivity and specificity for detecting osseous metastases [44,45]. Further, PET-CT is adept at excluding the potential presence of distant metastatic lesions, which has critical implications for patients who were originally considered to be surgical candidates [45].

### 2.4. Preoperative Biopsy

Preoperative biopsy plays a critical role in the multidisciplinary assessment of STS, as it allows for accurate tumor histological diagnosis as well as the establishment of an individualized treatment strategy [46]. In the case of retroperitoneal sarcomas, preoperative biopsy has not been found to negatively impact survival or increase local recurrence [47]. Conversely, improved outcomes, such as an increased likelihood of complete tumor resection, are associated with it [46]. Furthermore, preoperative core needle biopsy for retroperitoneal sarcoma has been demonstrated to be safe, with no need for hospitalization or early operation following the procedure [48].

Historically, incisional biopsy constituted the technique of choice for the histological diagnosis of soft-tissue masses. However, current evidence supports core needle biopsy as the standard of care for the assessment of STS. Compared to incisional biopsy, core needle biopsy has proven to convey similar diagnostic accuracy with less time between initial assessment and treatment recommendations [49]. Furthermore, ultrasound-guided core needle biopsy has proven to reliably predict histological tumor grade in extremity and trunk STS [50]. Additionally, CT-guided biopsy has been shown to be effective in assessing patients with visceral and retroperitoneal sarcomas [51]. According to current guidelines, core needle biopsy should be performed in sarcoma reference centers by experienced radiologists [26]. Moreover, higher diagnostic accuracy is attained by conducting an imaging-guided biopsy following a thorough MRI analysis, as it allows for comprehensive planning of the biopsy trajectory and adequate tissue targeting [26]. Due to the heterogeneous nature of the tumor, multiple cores using co-axial and 14–16 gauge needles should be obtained [52].

### 2.5. Radiation Therapy

External-beam radiation therapy plays a critical role in the local management of STS of the limbs and trunk. The use of RT largely depends on the risk assessment for local recurrence of STS [53]. In this respect, resection margin status constitutes the most important factor for predicting local recurrence [53]. Therefore, in the setting of STS of the limbs and trunk, the use of RT is strongly recommended in patients with an increased risk of local recurrence, particularly if close or microscopically positive margins are anticipated [53]. As outlined by the American Society for Radiation Oncology–Clinical Practice Guideline, current indications for RT in adult patients with STS of the extremities and trunk are summarized in Table 1.

Different timing strategies for the delivery of radiation therapy in patients with STS have been developed. Although similar rates of local control have been found between preoperative and postoperative RT [54], its distinctive applications have been outlined by established clinical practice protocols. Current guidelines recommend the delivery of RT in a preoperative manner for patients with primary, localized STS of the extremities and trunk [53]. Preoperative RT is strongly associated with acute local complications, particularly those related to wound healing [55]. In this respect, tumor size greater than 10 cm and tumor location in the lower extremities constitute important predictors of poor wound healing after preoperative RT [55]. Conversely, postoperative RT has been largely associated with long-term complications, particularly those related to limb function, such as fibrosis and joint stiffness [56]. Alternatively, the use of postoperative RT is indicated in patients with primary, localized STS in the extremities and trunk who underwent primary tumor resection and were, later, found to have unanticipated adverse features with increased risk of local recurrence [53]. Additional risks, regardless of the timing of RT, include the development of either bone or soft-tissue radiation-associated STS, which have a worse prognosis than their de novo cases [57], and radiation-associated fractures [58,59]. Current indications for preoperative and postoperative RT, as outlined by the American Society for Radiation Oncology—Clinical Practice Guideline, are summarized in Table 2. Critically, it is important to note that different histologic subtypes of STS have variable responses to RT; therefore, treatment with RT should be considered based on individual patient characteristics.

### 2.6. Chemotherapy

In the current literature, the role of chemotherapy in the management of STS remains controversial, with inconsistent findings across studies. Historically, chemotherapy was primarily used as palliative care for patients with advanced-stage disease. Nevertheless, with the advent of novel therapeutic approaches, the use of systemic therapies in the management of STS is gaining importance. Current guidelines emphasize the application of chemotherapy in selected clinical scenarios. In this regard, its use has been considered for patients with high-risk tumors and potentially more chemo-sensitive sarcoma subtypes, such as myxoid round cell sarcoma, synovial sarcoma, uterine leiomyosarcoma, and desmoplastic small round cell tumor [40]. Additionally, the role of chemotherapy has also been considered in clinical scenarios where local recurrence following therapy would be untreatable, and when the use of RT is not feasible due to the tumor’s proximity to sensitive structures [40]. The use of chemotherapy, as adjuvant or neoadjuvant treatment, has not been fully elucidated in the current literature [60]. Conventionally, chemotherapy has been primarily investigated as adjuvant therapy following surgical resection. Nevertheless, in spite of the high-quality studies conducted on the topic, conflicting findings regarding its efficacy as adjuvant therapy have been reported [15,16,17]. However, further investigation is required to fully elucidate its role. Currently, a combination of doxorubicin (also known as adriamycin) and ifosfamide has been considered the gold standard in the systemic management of STS [61]. However, innovative agents, such as gemcitabine, taxanes, and trabectedin, have been found to yield promising outcomes [61].

### 2.7. Alternative and Augmentative Treatment Approaches

There are significant challenges in the treatment of STS, which has prompted the development and investigation of various alternative and augmentative therapies, including cryosurgical ablation, immunotherapy, and regional hyperthermia. Cryosurgical ablation is a minimally invasive therapy that utilizes extreme cold to cause in-situ rapid freezing followed by slow thawing with several freeze-thaw cycles causing the devitalization of neoplastic tissue [62]. Phase 1 trials demonstrated promising early results, but further evaluation of efficacy is required [62]. Immunotherapy harnesses the immune system to target cancer cells. Approaches like immune checkpoint inhibitors and tyrosine kinase inhibitors in combination with chemotherapy and RT have shown promise in specific STS subtypes; however, the heterogeneous nature of STS limits its universal applicability [63]. Regional hyperthermia therapy exposes tumor cells to elevated temperatures of 40–43 °C, which has been found to work synergistically with chemotherapy and RT and has demonstrated improved survival when combined with standard treatments compared to chemotherapy alone [64]. While each modality offers unique advantages, their optimal integration into multidisciplinary treatment strategies is an ongoing focus of research aimed at improving efficacy and minimizing side effects.

## 3. Surgical Treatment

Wide surgical resection remains the gold standard for the treatment of localized STS. When performed, surgery seeks to achieve the following goals: (1) providing long-term survival and avoiding local recurrence, (2) maximizing function, and (3) minimizing morbidity. While negative surgical margins are a well-reported prognostic factor for local recurrence-free survival, there is conflicting data on the impact of margin status on overall and metastasis-free survival [65,66].

### 3.1. Margin Assessment

Current treatment guidelines do not provide a specific recommendation for what margin width constitutes a negative margin. Besides the width of the margin, assessment of margin adequacy should also consider the presence of anatomic barriers (e.g., fascia, periosteum), tumor histology, proximity of key neurovascular structures, and use of (neo)-adjuvant therapies [67]. Moreover, due to the rarity of STS, the majority of studies do not differentiate between infiltrative and non-infiltrative subtypes and use the same margin assessment tools indiscriminately [68].

The first classification system for surgical margins was described by Enneking et al. in 1980 and later adopted by the Musculoskeletal Tumor Society (MSTS) [69]. This system established 4 types of surgical margins based on the relationship of the tumor and its pseudocapsule to the margin: intralesional, marginal, wide, and radical (Table 3). In intralesional procedures, where the tumor pseudocapsule is breached, and in marginal resections, where the tumor is removed through the pseudocapsule or “reactive zone”, there is a risk of either macroscopic or microscopic residual tumor. Wide resections, which remove the tumor along with surrounding normal tissue within the involved compartment, minimize the risk of residual tumor and are the standard of care in STS surgery. Although radical resections, involving the removal of the entire anatomical compartment, may offer better local control, they are rarely performed due to their high morbidity and poor functional outcomes.

In recent years, newer classification schemes have been introduced. The most common are the R classification [70], the R + 1 mm classification [71], and the Toronto Margin Context Classification (Table 3) [72]. These systems improve upon the MSTS classification by distinguishing between microscopically and macroscopically positive margins, each with different prognosis implications. Additionally, some studies have relied on the dichotomous classification of margins as either positive or negative. Since a solely positive or negative margin status gives no insight between clear but close margins and broader margins, international guidelines recommend always reporting the distance between the tumor and surgical margin [73].

While the MSTS classification gained traction due to its simplicity and utility during surgical planning, reproducibility may be an issue. Trovik et al. reported 20% disagreement among a panel of sarcoma surgeons when deciding between marginal and wide-margin classification [74]. For both R classification and R + 1 mm classifications, the local recurrence rate of R0 resections is lower than that of R1 resections [75,76]. However, it remains unclear whether the R + 1 mm classification is superior to the R0 classification in predicting local recurrence. Ultimately, the margin assessment scheme used depends on the institution and surgeon performing the procedure.

While there are variable approaches to margin assessment, it should be noted that more extensive surgical margins may be recommended based on STS histology. Several STS histologic subtypes such as dermatofibrosarcoma, angiosarcoma, and myxofibrosarcoma infiltrate surrounding tissues and fascial planes through microscopic radial projections [77], Therefore, when anatomically feasible, a wide resection with a safety margin of up to 4 cm has been suggested to reduce the risk of recurrence after primary surgical resection [78].

### 3.2. Limb Salvage Versus Amputation

LSS has largely replaced amputation as the standard of care in the management of STS. This pivotal change was initially set by Rosenberg et al., whose work laid the groundwork for favoring limb preservation over radical amputation in STS [18]. In this respect, no significant differences were found regarding disease-free survival and overall survival in patients treated with amputation compared to those treated with LSS [11]. Recent advancements in surgical techniques have also prioritized limb function preservation as the main goal in STS management. Therefore, in cases where a functional limb cannot be preserved, such as extensive infiltration of a major nerve requiring resection, amputation followed by osseointegration offers superior functional outcomes compared to traditional amputation or limb-salvage with a non-functional extremity [79,80]. Additionally, targeted muscle reinnervation and regenerative peripheral nerve interfaces are valuable techniques in patients treated with full or partial amputation, as they allow for better control over myoelectric prostheses and phantom pain control [79]. Nevertheless, traditional amputation remains the optimal surgical technique in patients with STS who are not candidates for LSS (5 to 10%), as it enables adequate local control and symptomatic treatment [81]. Current indications for amputation in patients with STS of the limbs are as follows: anticipated inadequate limb function following R0 resection, multicompartmental neurovascular tumor spread, and local tumor seeding following unplanned surgery [82]. Other clinical scenarios where amputation may be a viable consideration include but are not limited to complications following LSS such as limb ischemia or infection, persistent local recurrence after repeated resection attempts, and in rare instances, palliative amputation may be considered appropriate [83].

### 3.3. Additional Surgical Consideration

#### 3.3.1. Periosteal Stripping

**Figure 1:**
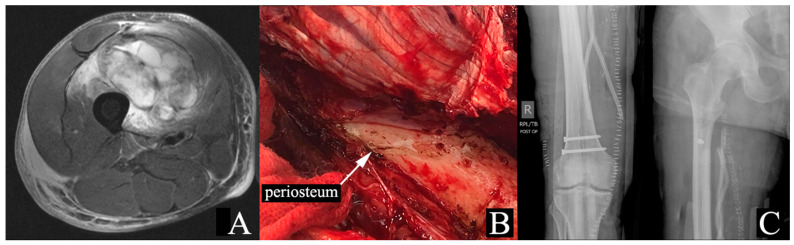
Seventy-one-year-old male with right thigh pleomorphic rhabdomyosarcoma. The patient was treated with 50 Gy of neoadjuvant RT. (**A**) MRI STIR sequence demonstrating proximity of the tumor to the femur, without invasion. (**B**) Intraoperative image showing periosteal stripping of the femur. (**C**) Plain radiographs displaying after periosteal stripping and RT. MRI: magnetic resonance imaging; STIR: Short Tau Inversion Recovery; RT: radiation therapy.

Excision of the periosteum (periosteal stripping) may be necessary to obtain a wide margin in STS that directly rests against the bone. While the periosteum can serve as an adequate margin, its excision disrupts the bone’s outer cortex vascularity and impairs bone healing [84,85]. In combination with RT, which also damages bone vascularity, patients are at significantly higher risk of pathologic fractures [86,87,88] (Figure 1). Lin et al. reported that all 9 patients who developed fractures in a cohort of 205 STS patients treated with surgery and RT had undergone periosteal excision [84]. The authors reported a 29% risk of fractures at 5 years among patients who had periosteal excision. Similarly, Helmstedter et al. found that 14 of 16 patients who developed fractures after surgery and RT for soft tissue tumors had periosteal stripping [87].

Unlike native bone fractures, fractures in previously irradiated bone have limited healing potential, with studies reporting a non-union rate of 44% to 82% for radiation-associated fractures [87,89]. In high-risk patients, including those with extensive periosteal stripping, prophylactic intramedullary nailing after sarcoma resection is recommended [87]. Additional risk factors to consider include age ≥ 65 years, tumor location in the anterior thigh compartment, higher radiation doses, and marginal or intralesional resection [87,90].

#### 3.3.2. Nerve Resection

Advances in imaging techniques and the use of neoadjuvant or adjuvant RT have expanded the indications for limb-salvage surgery after STS resection. With the increased use of skeletal and vascular reconstructions and the availability of rotational and free tissue transfers for large defects, major nerves now often define the limits of anatomic resection [91]. Traditionally, the involvement of a major motor nerve of the lower extremity was cited as an indication for amputation [92]. However, studies in the early 2000s showed that resection of major lower extremities resulted in acceptable functional deficits and was not an indication for amputation [93,94,95]. The technique of epineural dissection has further decreased the frequency of cases requiring complete major peripheral nerve resection to facilitate sarcoma excision [91,96]. Clarkson et al. found that using this technique in cases where nerve resection was anticipated to achieve negative margins achieved local recurrence rates comparable to those in patients whose tumors were distant from major nerves [96].

Currently, complete nerve resection is only necessary if the sarcoma invades the nerve itself. Careful epineural dissection often allows for the preservation of major nerves, leaving the nerve sheath as a margin [96]. In the few cases where nerve resection is required, nerve grafts, nerve transfers, and tendon transfers can enhance patient function [97]. Functional outcomes depend on the specific nerve involved, with resection of branches such as the peroneal and tibial nerves generally being better tolerated than resection of the sciatic or femoral nerves [91,93,94].

#### 3.3.3. Vascular Resection

Resection of major vessels, in a similar fashion to nerve resection, is often necessary to achieve wide margins. Arterial reconstruction is always indicated after limb salvage therapy due to the high risk of limb ischemia after arterial ligation [98]. Conversely, venous revascularization is not considered essential, as venous ligation does not compromise limb viability [98]. Vascular reconstruction is performed with either synthetic or autogenous vein grafts, with previous studies reporting a lower incidence of wound and graft infection with saphenous vein grafts [99,100]. Nishinari et al. reported higher occlusion rates with synthetic grafts compared to saphenous vein substitutes after vascular reconstruction in soft tissue sarcomas of the lower extremities [98]. Although patients undergoing vascular reconstruction are at higher risk for postoperative complications such as deep venous thrombosis, wound infection, and limb edema, their functional outcomes are not significantly worse than those of patients without vascular reconstruction [101]. When vascular reconstruction is planned, the impact of RT on the success of the vascular reconstruction should be considered. While studies on vascular reconstruction and RT are scarce, preoperative RT has been shown to reduce the viability of venous anastomosis in preclinical models and is an independent risk factor for complications after microvascular soft-tissue reconstructions [102,103].

#### 3.3.4. Soft Tissue Closure

As multidisciplinary management of STS becomes more prevalent, the literature increasingly highlights the benefits of plastic surgery involvement in soft tissue reconstruction [104]. Previous studies have shown that patients with vascularized tissue flaps experience lower complication rates, fewer secondary procedures, shorter hospital stays, and improved limb salvage rates compared to those who undergo primary wound closure [104,105].

**Figure 2:**
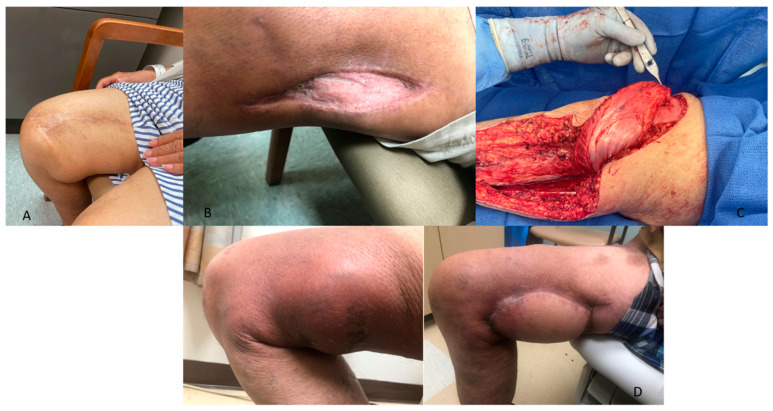
Multiple clinical images of patients with soft tissue sarcoma treated with soft tissue reconstruction. The reconstructive ladder was followed, performing procedures of increasing complexity, including (**A**) soft tissue rearrangement, (**B**) split-thickness skin graft, (**C**) rotational flap (gastrocnemius), and (**D**) myocutaneous free flap.

While the specific indications for complex soft tissue reconstruction using rotational or free flaps are beyond this review, a collaboration between orthopedic and plastic surgeons is critical for improving patient outcomes [106]. As part of an orthoplastic approach, preoperative discussions regarding the resection plan, anticipated skin and soft tissue removal, and use of RT are imperative for determining the need for complex reconstruction strategies (Figure 2).

## 4. Postoperative Period

### Postoperative Surveillance

The main objectives of postoperative surveillance programs in STS are early detection of local recurrences, detection of distant disease, new primary cancers, and treatment-related complications [107]. Nevertheless, due to the higher heterogeneity found among sarcoma subtypes, a standardized protocol for postoperative surveillance has not yet been widely established [108]. Standard surveillance includes assessing new signs or symptoms of local or distant recurrence, evaluating imaging needs based on clinical scenarios, and identifying treatment-related complications [40]. Chest imaging (radiography or CT) is an integral part of follow-up as pulmonary metastasis occurs in approximately 20% of STS patients at some point during their disease course [109]. Furthermore, the ACR Appropriateness Criteria recommends postoperative follow-up musculoskeletal imaging with MRI every 3–6 months during the first 10 years with consideration for spacing out to annual imaging between years 5–10 and additional imaging in the case of symptomatology [110]. For intermediate or high-grade sarcoma, follow-up is recommended every 3 to 4 months for the first 2 to 3 years, then every 6 months for up to 5 years, and, annually for 8 to 10 years [40]. For low-grade sarcomas, follow-up should occur every 6 months for 5 years and then annually [40]. Additionally, incorporating short-term ultrasonography in high-risk STS surveillance can enhance early detection of local recurrences and metastatic lymphadenopathy [111]. Tumor-specific surveillance protocols are also gaining importance as promising follow-up data for specific sarcoma subtypes emerges in the literature.

## 5. Complex Situations in STS Management

### 5.1. Previous Unplanned Excision

Unplanned excision refers to the macroscopic removal of malignant lesions without consideration for preoperative imaging, biopsy, or resection margins [112]. Patients with unplanned excisions most commonly present with small (<5 cm), superficial lesions that are mistaken for benign tumors [113,114]. Managing unplanned excisions is more complicated than treating primary STS due to potential tumor contamination of the previous surgical bed and distortion of normal tissue anatomy. While a history of unplanned excision is associated with higher local recurrence rates compared to planned resection, studies on its impact on patient survival yield conflicting results [115,116]. A recent meta-analysis by Larios et al. found that unplanned excision itself was not linked to worse overall survival but was a risk factor for local recurrence, which correlated with poorer overall survival [117]. Due to the need to remove extensive scar tissue and the distorted anatomy and tissue architecture, the product of previous surgery, unplanned excisions are also associated with higher rates of plastic reconstructive surgery compared to planned resections [118].

Due to the unreliability of the reported margins in the initial surgery, and the residual disease rates of 24% to 91% at re-excision [115,116,119], tumor bed excision is recommended in most cases of unplanned excision. Certain authors have suggested postponing re-excision until local recurrence occurs, based on the assumption that local control has no impact on overall survival [120]. Bonvalot et al., citing a lack of correlation between local recurrence and overall survival, recommended a “wait and see” approach, performing tumor bed excision only in cases of tumor rupture or gross residual disease [120]. However, a recent meta-analysis by Larios et al. found that unplanned excision increases the risk of local recurrence (relative risk [RR] = 1.35), which in turn negatively affects 5-year overall survival (RR = 1.82) [117]. Although unplanned excision did not directly impact survival, the increased local recurrence risk underscores the importance of re-excision to remove residual disease. Without clear evidence to suggest that residual disease is not prognostic in unplanned excisions—contradicting current literature—not performing a re-excision would be difficult for both the surgeon and patient to accept.

### 5.2. Microscopically Invasive Tumors

Resection with negative margins is the most important predictor of local recurrence, a highly morbid and potentially fatal oncologic outcome [121]. To reduce this risk, surgeons use intraoperative frozen pathology and gross examination to evaluate surgical margins [122,123]. However, these methods can be unreliable in cases of microscopically invasive tumors, such as myxofibrosarcoma, and dermatofibrosarcoma protuberans leading to false negative readings and an increased risk of local recurrence [117,124]. While final pathology is the *gold standard* for margin assessment, results often take more than a week to be available and surgeons may opt for performing single-stage resection/reconstruction and postoperative RT. However, even with the postoperative RT, local tumor control is compromised due to positive surgical margins and microscopic tumor extension beyond the irradiated field [125].

In response to the inherent challenges of myxofibrosarcoma resections, authors have suggested temporizing the wound bed with vacuum-assisted closure (VAC) after surgery, while awaiting formal margin analysis [126,127]. Upon final results, patients with positive margins can be taken back to the OR for tumor bed re-resection of the areas of margin positivity. Once all margins are negative on the final pathology assessment, final soft-tissue coverage is performed. The timing of radiotherapy (RT) in conjunction with wound VAC temporization varies across institutions; some perform preoperative RT followed by VAC temporization, while others opt for postoperative RT only after VAC temporization and achieve negative margins. Fourman et al. compared wound VAC temporization to single-stage resection/reconstruction, and found lower R1 resection and local recurrence rates [127]. While this strategy requires additional surgical procedures and potentially longer lengths of stay, further studies have reported similar patient-reported outcomes and cost profiles compared to single-stage resection/reconstruction [128,129,130]. Additionally, the use of fluorescence-guided, which can provide real-time knowledge of margin status after performing a wide resection, may further diminish local recurrence rates in these tumors [131].

### 5.3. Tumor Recurrence

a. Local recurrence

Local recurrence rates after resection with positive margins of STS are reportedly 80% to 90% [132,133]. However, newer imaging techniques, the use of adjuvant and neoadjuvant RT, and appropriate resection techniques have diminished local recurrence rates to 7% to 15% [134,135]. Management of local recurrence is inherently complex, as patients are reportedly predisposed to future recurrences [136]. Prior treatment with RT and surgery further complicates the management of local recurrences, and the best strategy remains unclear.

The optimal treatment strategy hinges on accurately determining whether the local recurrence is resectable. For resectable STS recurrences, surgical re-excision, with or without concomitant RT or systemic treatment, is recommended [12]. The use of RT to treat the recurrence depends on prior treatment, as reirradiation is associated with significant toxicity [55]. Torres et al. reported that in 62 patients with locally recurrent STS treated with wide local re-excision after prior surgery and RT, reirradiation was not associated with improved local control [137]. However, the authors found higher complication (80% vs. 17%) and amputation rates (35% vs. 11%) in patients treated with surgery and reirradiation compared to those treated with surgery alone [137]. When reirradiation is considered, proton therapy is recommended due to the higher tolerability and lower associated toxicities [138]. The use of concomitant chemotherapy for resectable STS recurrences remains controversial, with multiple meta-analyses showing conflicting results on its impact on overall survival [15,16]. Ultimately, treatment of local recurrences varies by institution, with wide re-excision as the cornerstone. For unresectable lesions or disseminated metastatic disease, regional therapies like isolated limb perfusion or infusion [139], combined with palliative chemotherapy, are typically indicated. Obtaining a preoperative MRI of the soft tissue mass is of critical importance for surgical planning as features such as proximity to vascular structures [140], peritumoral enhancement [141], and “tail sign” [142] can impact the treatment approach; thereby, also affecting the likelihood of local recurrence.

b. Distant recurrence

STS are typically diagnosed at early stages, with only 14% of patients presenting with metastatic disease [143]. However, an additional 40 to 50% of patients will develop metastases within 5 years [6]. All patients with metastatic STS should be discussed at a multidisciplinary tumor board, with tumor histology and extent of disease as the main drivers of management. Unlike other tumors, metastatic STS is often restricted to a single organ, with 70% of patients presenting metastasis limited to the lungs [144]. The concept of oligometastatic disease, characterized by fewer than five metastases in a single organ or limited organs, has recently gained recognition as a distinct stage between localized and widespread metastatic disease. Notably, multiple studies have shown long-term survival after resection of isolated pulmonary nodules in select patients with oligometastatic STS [145,146]. Prognostic factors for improved survival after lung metastasectomy include adequate tumor resection, disease-free interval > 12 months, unilateral pulmonary metastasis, and metachronous metastases [144]. Besides lung metastases, isolated hepatic metastases may also be amenable to curative resection, although data is conflicting. In patients who are not candidates for surgical metastasectomy, stereotactic body RT and local ablative procedures are also effective strategies for local tumor control in metastatic sites [147]. For patients with disseminated metastatic disease, cytotoxic chemotherapy is the mainstay treatment [143]. However, the use of histology-directed treatment for certain subtypes of STS that are highly sensitive to targeted therapy but insensitive to conventional chemotherapy should be considered.

## 6. Conclusions

The management of STS has become a multidisciplinary process integrating advances in preoperative assessment, imaging, surgical techniques, and adjuvant therapies. Thorough preoperative evaluation, particularly individualized imaging and biopsy is essential for effective treatment planning. Surgical resection with negative margins remains the cornerstone of STS management, while adjuvant therapies, including RT and chemotherapy, play supportive roles in selected cases to minimize local recurrence and improve survival rates.

Emerging techniques in LSS, combined with developments in nerve and vascular reconstruction, have shifted the focus toward preserving function without compromising oncologic control. As a result, limb-salvage techniques have largely replaced amputation. Vigilant postoperative monitoring and follow-up are crucial to managing recurrence risks. This comprehensive, evidence-based approach ensures tailored care for STS patients. Ongoing research into imaging, chemotherapy, and targeted therapies will refine management strategies further, especially in complex and recurrent cases. Ultimately, this review highlights the essential aspects of STS management and underscores the need for coordinated, multidisciplinary care to enhance both survival and quality of life for affected patients.

## Figures and Tables

**Table 1:** Indications for RT in the treatment of extremity and superficial truncal STS in adults, according to the ASTRO Clinical Practice [53].

	Recommendations	Strength of Recommendation
1	For patients with primary, localized extremity and truncal soft-tissue sarcoma (STS) for whom oncologic resection is planned, RT is recommended for those at increased risk for local recurrence.	Strong
2	For patients with primary, localized extremity and truncal STS for whom oncologic resection is planned and a close or microscopically positive margin is anticipated, RT is recommended.	Strong
3	For patients with primary, localized extremity and truncal STS for whom oncologic resection is planned, RT is not recommended for those at low risk for local recurrence.	Strong

ASTRO: American Society for Radiation Oncology; RT: radiotherapy; STS: soft tissue sarcoma.

**Table 2:** Indications for preoperative and postoperative RT in the treatment of extremity and superficial truncal STS in adults, according to the ASTRO Clinical Practice Guideline [53].

		Recommendations	Strength of Recommendation
1		For patients with primary, localized extremity and truncal STS, the sequencing of surgery and RT should be determined based on multidisciplinary evaluation of patient and tumor characteristics.	Strong
2	Preoperative RT	For patients with primary, localized extremity and truncal STS, where surgery and RT are indicated, preoperative RT is recommended over postoperative RT.	Strong
3	Postoperative RT	For patients with primary, localized extremity and truncal STS treated with initial oncologic resection (without preoperative RT) found to have unanticipated adverse pathologic features, postoperative RT is recommended.	Strong
4		For patients with primary, localized extremity and truncal STS, where surgery and RT are indicated, initial oncologic resection followed by postoperative RT is conditionally recommended in specific clinical circumstances (e.g., uncontrolled pain or bleeding, fungating tumors), or when the risk of wound healing complications outweighs that of late toxicity.	Conditional

ASTRO: American Society for Radiation Oncology; RT: radiotherapy; STS: soft tissue sarcoma.

**Table 3:** Classification systems for surgical margins in STS.

MSTS [69]			AJCC (R Classification) [70]	UICC (R+1 mm Classification) [71]	TMCC [72]	
**Margin Class**	**Plane of Dissection**	**Margin Class**	**Description**		**Margin Class**	**Description**
**Radical**	Extracompartmental *en bloc* entire compartment	**R0**	Tumor does not reach intact barrier or resection margins	Resection margin > 1 mm	**Negative**	Tumor does not reach intact barrier or resection margins
**Wide**	Intracompartmental *en bloc* with cuff of normal tissue	**R1**	Microscopic residual tumor	Resection margin < 1 mm	**Planned closure**	Positive surgical margins against one or more critical structures (nerve, vessel or bone) which had been planned preoperatively as part of a primary resection
**Marginal**	Shell out *en bloc* through pseudocapsule or reactive zone	**R2**	Macroscopic residual tumor	Macroscopic tumor contamination	**Positive after prior unplanned excision**	Positive margin on tumor bed re-excision
**Intralesional**	Piecemeal debulking or curettage				**Unplanned positive margins**	Positive margin which had not been planned

*AJCC*: American Joint Committee on Cancer (R classification); *MSTS*: Musculoskeletal Tumor Society; *UICC*: Union for International Cancer Control; *TMCC*: Toronto Margin Context Classification.
