# Correction: Gonzalez et al. Principles of Surgical Treatment of Soft Tissue Sarcomas. *Cancers* 2025, *17*, 401

**DOI:** 10.3390/cancers17132096

**Published:** 2025-06-23

**Authors:** Marcos R. Gonzalez, Carolina Mendez-Guerra, Megan H. Goh, Juan Pretell-Mazzini

**Affiliations:** 1 Division of Orthopaedic Oncology, Department of Orthopaedic Surgery, Massachusetts General Hospital, Harvard Medical School, Boston, MA 02115, USA; mgonzalez52@mgh.harvard.edu (M.R.G.); mgoh3@mgh.harvard.edu (M.H.G.); 2 Facultad de Ciencias de la Salud, Universidad Peruana de Ciencias Aplicadas, Lima 15067, Peru; mendezguerra.ci@gmail.com; 3 Division of Orthopedic Oncology, Miami Cancer Institute, Baptist Health System South Florida, Plantation, FL 33324, USA; 4 Department of Orthopaedic Surgery, Herbert Wertheim College of Medicine, Florida International University, Miami, FL 33199, USA

In the original publication [1], there was a mistake: Table 3 and its citations in the main text were missing. The correct Table 3 and its caption appear below. The citations for Table 3 have been added in *3.1. Margin Assessment*.

The authors state that the scientific conclusions are unaffected. This correction was approved by the Academic Editor. The original publication has also been updated.

## Figures and Tables

**Table 3:** Classification systems for surgical margins in STS.

MSTS [69]			AJCC (R Classification) [70]	UICC (R + 1 mm Classification) [71]	TMCC [72]	
**Margin Class**	**Plane of Dissection**	**Margin Class**	**Description**		**Margin Class**	**Description**
**Radical**	Extracompartmental *en bloc* entire compartment	**R0**	Tumor does not reach intact barrier or resection margins	Resection margin > 1 mm	**Negative**	Tumor does not reach intact barrier or resection margins
**Wide**	Intracompartmental *en bloc* with cuff of normal tissue	**R1**	Microscopic residual tumor	Resection margin < 1 mm	**Planned closure**	Positive surgical margins against one or more critical structures (nerve, vessel or bone) which had been planned preoperatively as part of a primary resection
**Marginal**	Shell out *en bloc* through pseudocapsule or reactive zone	**R2**	Macroscopic residual tumor	Macroscopic tumor contamination	**Positive after prior unplanned excision**	Positive margin on tumor bed re-excision
**Intralesional**	Piecemeal debulking or curettage				**Unplanned positive margins**	Positive margin which had not been planned

*AJCC*: American Joint Committee on Cancer (R classification); *MSTS*: Musculoskeletal Tumor Society; *UICC*: Union for International Cancer Control; *TMCC*: Toronto Margin Context Classification.
